# Assessing SNP-SNP Interactions among DNA Repair, Modification and Metabolism Related Pathway Genes in Breast Cancer Susceptibility

**DOI:** 10.1371/journal.pone.0064896

**Published:** 2013-06-03

**Authors:** Yadav Sapkota, John R. Mackey, Raymond Lai, Conrado Franco-Villalobos, Sasha Lupichuk, Paula J. Robson, Karen Kopciuk, Carol E. Cass, Yutaka Yasui, Sambasivarao Damaraju

**Affiliations:** 1 Cross Cancer Institute, Alberta Health Services, Edmonton, Alberta, Canada; 2 Department of Laboratory Medicine and Pathology, University of Alberta, Edmonton, Alberta, Canada; 3 Department of Oncology, University of Alberta, Edmonton, Alberta, Canada; 4 School of Public Health, University of Alberta, Edmonton, Alberta, Canada; 5 Department of Oncology, University of Calgary, Calgary, Alberta, Canada; 6 Department of Agricultural, Food and Nutritional Sciences, University of Alberta, Edmonton, Alberta, Canada; 7 Department of Mathematics and Statistics, University of Calgary, Calgary, Alberta, Canada; 8 Alberta Health Services – Cancer Care, Edmonton, Alberta, Canada; 9 Tom Baker Cancer Centre, Alberta Health Services, Calgary, Alberta, Canada; Dartmouth, United States of America

## Abstract

Genome-wide association studies (GWASs) have identified low-penetrance common variants (*i.e.*, single nucleotide polymorphisms, SNPs) associated with breast cancer susceptibility. Although GWASs are primarily focused on single-locus effects, gene-gene interactions (*i.e.*, epistasis) are also assumed to contribute to the genetic risks for complex diseases including breast cancer. While it has been hypothesized that moderately ranked (*P* value based) weak single-locus effects in GWASs could potentially harbor valuable information for evaluating epistasis, we lack systematic efforts to investigate SNPs showing consistent associations with weak statistical significance across independent discovery and replication stages. The objectives of this study were i) to select SNPs showing single-locus effects with weak statistical significance for breast cancer in a GWAS and/or candidate-gene studies; ii) to replicate these SNPs in an independent set of breast cancer cases and controls; and iii) to explore their potential SNP-SNP interactions contributing to breast cancer susceptibility. A total of 17 SNPs related to DNA repair, modification and metabolism pathway genes were selected since these pathways offer *a priori* knowledge for potential epistatic interactions and an overall role in breast carcinogenesis. The study design included predominantly Caucasian women (2,795 cases and 4,505 controls) from Alberta, Canada. We observed two two-way SNP-SNP interactions (*APEX1*-rs1130409 and *RPAP1*-rs2297381; *MLH1*-rs1799977 and *MDM2*-rs769412) in logistic regression that conferred elevated risks for breast cancer (*P*_interaction_<7.3×10^−3^). Logic regression identified an interaction involving four SNPs (*MBD2*-rs4041245, *MLH1*-rs1799977, *MDM2*-rs769412, *BRCA2*-rs1799943) (*P*_permutation_ = 2.4×10^−3^). SNPs involved in SNP-SNP interactions also showed single-locus effects with weak statistical significance, while *BRCA2*-rs1799943 showed stronger statistical significance (*P*_correlation/trend_ = 3.2×10^−4^) than the others. These single-locus effects were independent of body mass index. Our results provide a framework for evaluating SNPs showing statistically weak but reproducible single-locus effects for epistatic effects contributing to disease susceptibility.

## Introduction

Breast cancer is a multifactorial disease, which results from combined effect of genetic, reproductive, environmental, and lifestyle risk factors. Linkage and twin studies revealed familial clustering of breast cancer, giving an approximately two-fold higher risk for first-degree relatives with family history [1], [2]. Although some familial clustering is explained by germline mutations in high or moderate penetrance genes such as *BRCA1* [3], *BRCA2* [4], *ATM* [5], *PTEN* [6], *TP53* [7], *BRIP1* [8], *PALB2* [9] and *CHEK2* [10], such mutations are rare in the general population [11]–[13]. Hence, a polygenic model has been proposed to explain the bulk of genetic susceptibility in sporadic and non-*BRCA* breast cancers [13]. Under this model, a combination of multiple low penetrance loci/genes across the genome would contribute to overall genetic risk.

Several genome-wide association studies (GWASs) identified multiple single nucleotide polymorphisms (SNPs) statistically significantly associated with breast cancer susceptibility [12], [14]–[21], supporting the polygenic model. However, these low penetrance variants, together with known predisposition genes (*e.g., BRCA1* and *BRCA2*), explain only a small proportion of the total genetic risk of breast cancer [16], suggesting that more variants exist. Identifying additional low penetrance variants is difficult because the effect size is expected to be smaller than the GWAS variants reported thus far, requiring large sample sizes. Collaborative efforts are now underway from international consortia to profile additional low penetrance variants. Current GWAS approaches largely rely on single-locus effects of SNPs with the disease of interest, studied one SNP at a time, while ignoring potential SNP-SNP interactions at two or more loci (*i.e.*, epistatic effects) [22], [23]. Epistasis is a ubiquitous phenomenon that describes how genes/loci interact to affect phenotypes. Such interactions are assumed to contribute to breast cancer. In search of the putative genes or SNPs contributing to epistasis, we reasoned that a study design exclusively addressing the value of GWAS or candidate gene SNPs with single-locus effects with weak statistical significance (hereafter referred to as “weak single-locus effects”) but with acting within a common biological pathway would provide mechanistic support for such a premise, which otherwise might be overlooked in less constrained genetic association studies. There is support to the premise that SNPs with weak single-locus effects are indeed of value to explore for epistatic effects, which in turn may contribute to a substantial proportion of the overall heritable risk [24], [25]. While GWAS approaches are still crucial to initially scan the genome and to identify variants with appreciable single-locus effects, further analyses capturing the combined effects of two or more SNPs with weak but reproducible single-locus effects in independent stages/studies may shed light into unexplained heritability of breast cancer.

Recently, we conducted a two-stage association study using SNPs selected from GWAS for sporadic breast cancer [26]. The SNPs selected were located in or close to DNA repair, modification and metabolism pathway related genes and showed weak single-locus effects for breast cancer [26]. In a combined sample size of 1,480 breast cancer cases and 1,635 apparently healthy controls from two independent stages, we observed six SNPs (located on chromosomes 8, 10, 15 and 18) showing weak but consistently reproducible single-locus effects for breast cancer susceptibility (per allele odds ratio (OR) ranged 0.85–0.86 for three protective SNPs and 1.13–1.20 for three risk elevating SNPs). We hypothesized that these variants may be optimal candidates to investigate potential SNP-SNP interactions at two or more loci contributing to breast cancer etiology.

To enable a more comprehensive evaluation of epistatic interactions among SNPs, we also considered additional SNPs from cancer related DNA repair genes, with prior evidence of their weak single-locus effects for breast cancer [27]–[35]. Genetic variations in DNA repair genes are extensively studied in the context of breast cancer as inter-individual variations in DNA repair capacity has been ascribed to contribute to heritable component of breast cancer [11], [36]. Despite large efforts by investigators/consortia, DNA repair genes/loci identified from GWASs that contribute to breast cancer susceptibility are limited. This further strengthens the premise that DNA repair related SNPs may potentially contribute through the epistatic mechanism. The bulk of the literature from biochemical characterizations of DNA repair proteins indicate that these gene products are involved in protein-protein and DNA-protein interactions to repair damage to DNA by carcinogens and radiation induced effects. To our knowledge, this is the first study attempting to assess potential SNP-SNP interactions at two or more loci implicated in breast cancer susceptibility, using systematically selected SNPs based on functional criteria from both GWAS and candidate gene approaches. Furthermore, we also investigated the single-locus effects of SNPs considered in this study to examine their reproducibility in an independent study population before assessing their potential epistatic effects, while adjusting for body mass index (BMI), a known risk factor for breast cancer.

## Materials and Methods

### Study Participants

Breast cancer cases (n = 2,795) used in this study were accessed from the provincial tumor bank located at the Cross Cancer Institute, Edmonton, Alberta, Canada (http://www.abtumorbank.com/), and the description of these has been described in detail elsewhere [19], [26]. This tumor bank contains well-annotated clinicopathological characteristics of the samples stored. The breast cancer cases included in this study had a pathologically confirmed diagnosis of invasive breast cancer predominantly characterized by late onset of disease (*i.e.*, median age and range at diagnosis = 54 and 21–92 years, respectively, with >92% of the cases aged 40+ years at the time of diagnosis). The median BMI of breast cancer cases at the time of diagnosis was 27.4 and range 15.6–62.3. Apparently healthy controls (n = 4,505) were accessed from the Tomorrow Project (http://in4tomorrow.ca/) [19], [26], Edmonton, Alberta, Canada, which aims to capture lifestyle factors and DNA of approximately 50,000 healthy Albertans enrolled in the prospective cohort study. The median age and range at blood draw were 54 and 34–78 years, respectively, with >92% of the controls aged 40+ years at the time of blood draw. The median BMI of healthy controls at the time of enrollment in the study was 25.5 and range 10.4–60.4. The breast cancer cases and controls in this study were predominantly of Caucasian origin based on their self-declared ethnicity and the overall demographics of the region. Written informed consents were obtained from all the study participants and the study was approved by the Alberta Cancer Research Ethics Committee, Alberta, Canada.

### SNPs and Samples Considered

**Figure 1:**
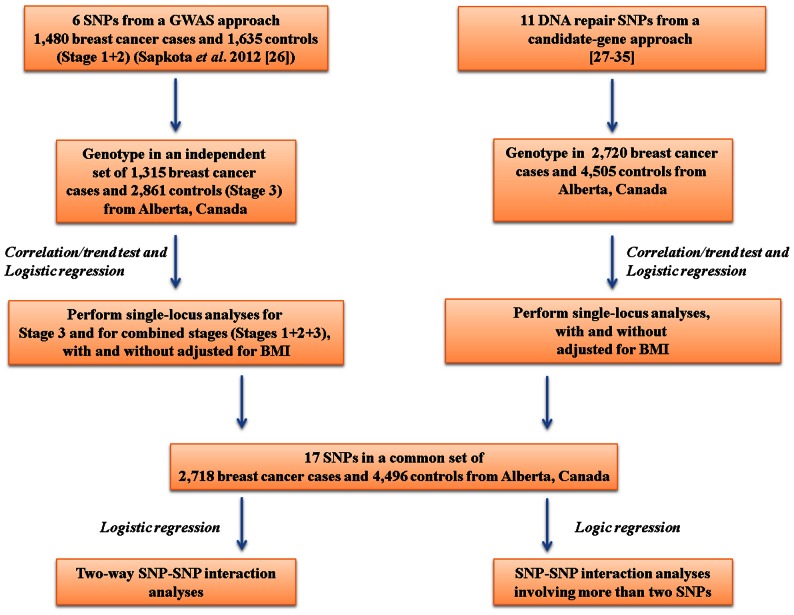
An overview of the study design.

A total of 17 candidate SNPs located in or close to 14 DNA repair, modification and metabolism pathway related genes (*RAD21, MGMT*, *RPAP1, MBD2, PARP1, MLH1, MSH3, ERCC6, MDM2, BRCA2, ERCC5, APEX1, XRCC3* and *XRCC1*) were considered (**Text S1 and Tables S1 and S2**). Of these, six SNPs (8q24.11-rs13250873, 10q26.3-rs1556459, *RPAP1*-rs2297381, *MBD2*-rs7614, *MBD2*-rs4041245 and *MBD2*-rs8094493) were selected from GWAS and previously replicated in an independent set of breast cancer cases and healthy controls [19], [26]. These SNPs were genotyped as part of a stage 3 study in additional breast cancer cases (n = 1,315) and healthy controls (n = 2,861) and were evaluated for their single-locus effects for breast cancer (**Text S1** and **Table S1**). Overall, we present our findings from a combined sample size of 2,795 breast cancer cases and 4,496 controls from all three stages to meet the statistical rigor. The remaining 11 candidate DNA repair SNPs (*PARP1*-rs1136410, *MLH1*-rs1799977, *MSH3*-rs184967, *MSH3*-rs26279, *ERCC6*-rs2228528, *MDM2*-rs769412, *BRCA2*-rs1799943, *ERCC5*-rs17655, *APEX1*-rs1130409, *XRCC3*-rs1799796 and *XRCC1*-rs25487) were selected based on published DNA repair gene polymorphisms and their associations with breast cancer susceptibility [27]–[35], our pilot study screening for more than 100 SNPs from 59 genes showing high minor allele frequency, concordance of genotypes to Hardy-Weinberg Equilibrium (HWE) in controls, statistical significance for the association in overall case-control analysis or promising associations (allelic and/or genotypic) for subtypes of breast cancer addressing the inherent heterogeneity, and high SNP call rates (data not shown). These 11 SNPs were genotyped in 2,720 breast cancer cases and 4,505 controls and were evaluated for their single-locus effects for breast cancer. To evaluate SNP-SNP interactions, we used genotype data of the 17 SNPs represented in a common set of breast cancer cases (n = 2,718) and healthy controls (n = 4,496). The finite discrepancies between the numbers of samples used for genotyping of the profiled SNPs and those used for SNP-SNP interactions were expected due to multiplexing assays for SNPs and the panels designed for the genotyping experiments on the Sequenom iPLEX Gold platform. An overview of the study design is presented in **Figure 1**.

### SNP Genotyping and Quality Control

Genotyping assays of the 17 SNPs were designed and performed on the Sequenom iPLEX Gold platform (San Diego, CA, USA) using services from the McGill University and Genome Quebec Innovation Center, Montreal, Canada. Genotype concordance among SNPs was assessed using 66 duplicate samples (8 cases and 58 controls). Thresholds for SNP call rates of >99% and HWE *P*>10^−6^ in controls were adopted.

### Statistical Considerations

We evaluated potential interactions among the select 17 candidate SNPs at two loci using logistic regression and multiple loci using logic regression. Logistic regression models using the command ‘-*epistasis*’ in PLINK (http://pngu.mgh.harvard.edu/~purcell/plink/) [37] were used to assess two-way interactions and reported as ORs, 95% confidence intervals (CIs) and *P* values associated with the b3 coefficient of the following model:
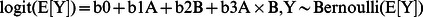
where b3 captures the two-way interaction between SNP A and SNP B. To correct for multiple comparisons, we calculated the Benjamini-Hochberg False Discovery Rate (FDR) [38].

Logic regression is a method to assess SNP-SNP interaction among multiple loci, and it has been successfully applied to a GWAS SNP data recently [39], in addition to a candidate-gene approach [40]. Logic regression searches for a set of predictors that are Boolean combinations of binary SNP covariates using intersection (“AND”) and union (“OR”) operations. To explore potential multi-way SNP-SNP interactions among the 17 SNPs considered in this study, we fitted a logic regression model using the *LogicReg* package [41] available in R 2.15.1 [42]. We excluded 157 (2.1%) subjects due to missing genotype as the *LogicReg* do not allow missing data. Since SNPs can have three possible genotypes (*e.g.*, AA, AB, BB), we first recoded the 17 SNPs into two sets of binary covariates by using both dominant (*e.g.,* AA = 1, AB = 1, BB = 0) and recessive (*e.g.,* AA = 0, AB = 0, BB = 1) and fitted the logic regression of the following form:
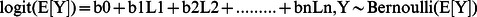
where L_i_ is a Boolean combination of the binary SNP covariates such as [(*SNP A = AA OR SNP B = AA) AND SNP C = AB or BB*], also known as a logic tree. A score function (deviance of the model) was then used to evaluate models with the number of trees, *n*, in the range of [2], [5] and the total number of SNPs in the range of [2], [17] using a 10-fold cross validation approach to determine the optimal tree/SNPs size. We evaluated the statistical significance of a final model with the optimal tree/SNPs size using a permutation test with 10,000 permutations of the case control labels. All statistical tests were two-sided.

## Results

Genotyping assays for each of the 17 SNPs were successful with a SNP call rate of >99% and the SNPs also passed HWE (*P*>10^−6^) in controls (**Tables S1 and S2**). Average genotype concordance was 100% for the 17 SNPs. Single-locus association tests in independent stages or in combined stage, adjusted for BMI were also profiled. Overall, SNPs considered in this study conferred weak single-locus effects for breast cancer, as we expected. We also analyzed the SNP-breast cancer associations by removing subjects from cases and controls with extreme ages (<35 yrs. and >80 yrs.) and BMI (<18.5 and >40). The associations did not change materially, suggesting that the small fraction (∼6.4% or 468 subjects) of extreme subjects may not have modified the observed overall SNP-breast cancer associations, data not shown.

### Two-way SNP-SNP Interactions

Logistic models were used to assess all SNP pairs among 17 candidate SNPs. Of these, two SNP pairs (*APEX1*-rs1130409 *RPAP1-rs2297381 and *MLH1*-rs1799977 *MDM2-rs769412) showed the strongest statistical association with breast cancer (*P*<7.3×10^−3^), with modest FDR values of 0.30 and 0.49, respectively (**Table 1**). Both SNP pairs showed increased risks towards breast cancer with ORs and 95% CIs of 1.16 [1.06–1.28] and 1.33 [1.08–1.64], respectively. The observed risks were similar for cases with luminal A tumors (∼70% of the total cases), while the interactions were not statistically significant when analyses were restricted to cases with luminal B, HER2+ and triple negative tumors, data not shown.

**Table 1:** Two-way interactions identified among DNA repair pathway related SNPs.

SNP1×SNP2	ORs [95% CI]	*P* *	FDR**
*APEX1*-rs1130409 * *RPAP1*-rs2297381	1.16 [1.06–1.28]	2.25E-03	0.30
*MLH1*-rs1799977 * *MDM2*-rs769412	1.33 [1.08–1.64]	7.31E-03	0.49

* *P* values obtained from unconditional logistic regression models.

** Corrected for multiple comparisons using Benjamini-Hochberg False Discovery Rate method.

### SNP-SNP Interactions Involving Multiple SNPs Using Logic Regression

Logic regression including the 17 SNPs identified a logic structure representing a SNP-SNP interaction involving four SNPs and was statistically significant (*P* = 2.4×10^−3^) (**Table 2**). The logic structure contained two logic trees, one with three SNPs and another with one SNP. The first logic tree consisted of an intersection of a union of *MBD2*-rs4041245 and *MLH1*-rs1799977 and *MDM2*-rs769412 while the second logic tree contained *BRCA2*-rs1799943. These logic trees formed four logic-based risk groups; a reference group (OR = 1.00) and two low risk groups, with ORs 0.79 and 0.90, respectively and a high risk group with OR 1.18. The observed logic structure was tested in subgroups of tumors. It was statistically significant for the subgroup of cases with luminal A tumors (*P* = 3.3×10^−3^), while it was not in other subgroups (luminal B, HER2+ and triple negatives tumors, data not shown).

**Table 2:** Multi-way SNP-SNP interactions identified by logic regression.

		rs4041245		rs1799977	rs769412	rs1799943	Logic-based Risk Groups		
		AA		AA	AA	AA/AG			
Genotype Frequency	Cases	952		1329	2373	1306			
	N = 2662	(35.77%)		(49.92%)	(89.14%)	(49.06%)			
	Controls	1405		2071	3845	1990			
	N = 4395	(31.97%)		(47.12%)	(87.49%)	(45.28%)			
Logic 1		(OR )	(AND )				Frequency		
Logic 2							Cases	Controls	Odds Ratio
Logic-based Risk Groups		Logic 1 = No				Logic 2 = No	527	1076	0.79
		Logic 1 = Yes				Logic 2 = No	829	1329	1.00
		Logic 1 = No				Logic 2 = Yes	500	895	0.90
		Logic 1 = Yes				Logic 2 = Yes	806	1095	1.18

## Discussion

In this study of more than seven thousand women, we evaluated the contribution of epistasis to breast cancer susceptibility among 17 SNPs located in or close proximity to 14 DNA repair, modification and metabolism pathway related genes. We identified two SNP pairs and interactions involving four SNPs among seven candidate SNPs located in seven genes. Except for *APEX1*-rs1130409, the SNPs participating in SNP-SNP interactions also showed weak single-locus effects (both allelic and genotypic) for breast cancer, independent of BMI (**Text S1 and Tables S1 and S2**). Of these, *BRCA2*-rs1799943 showed the strongest single-locus effects. Overall, our findings support the notion that SNPs with reproducible weak single-locus effects are useful candidates for studying their potential epistatic effects contributing to breast cancer susceptibility.

We identified two SNP pairs that demonstrated significant interactive effects on breast cancer risk and carried modest FDR values. Of these, one was *MBD2* SNP we reported earlier and the other three were from the candidate DNA repair SNPs considered in this study. The first pair consisted of *APEX1*-rs1130409 and *RPAP1-*rs2297381, with an OR of their interaction as 1.16, which was greater than their individual single-locus effects of 1.01 and 1.07, respectively. Similarly, another pair included *MLH1*-rs1799977 and *MDM2*-rs769412, with an OR of their interaction as 1.33 conferring risk. Interestingly, their individual single-locus effects were in opposite direction with ORs of 0.94 and 0.86, respectively and deserve further independent replication of findings.

Using a logic regression model, we also detected SNP-SNP interactions involving four SNPs (*MBD2*-rs4041245, *MLH1*-rs1799977, *MDM2*-rs769412 and *BRCA2*-rs1799943). Interestingly, one of the SNPs we entered in this analysis and was predicted to participate in epistatic effects from a previous study [26] was also identified to partner with three other SNPs we profiled from the DNA repair genes considered in this study. Except for *MLH1*-rs1799977 and *MDM2*-rs769412, this model captured distinct set of SNPs from the ones profiled in the two-way epistatic interactions, suggesting a possible convergence of multiple DNA repair pathways while conferring breast cancer risk. Future independent studies through large international consortia are warranted to further evaluate the contributions of the observed SNP-SNP interactions to breast cancer predisposition. We believe these findings reflect important biology, rather than simply statistical artifacts because of the unprecedented amount of literature indicating DNA-protein and protein interactions involved in DNA repair process.

We further investigated for possible biological insights in to the observed SNP-SNP interactions using a Cytoscape plugin, GENEMANIA [43]. For a given set of genes, GENEMANIA predicts their functional relationships, such as genetic and protein interactions, pathways, co-expressions, co-localization and similar protein domains from mining publicly available knowledgebase (*e.g.*, PubMed, BioGRID, PathwayCommons and Pfam). We observed that the SNP-SNP interactions we identified were also complimented by observed/predicted interactions among the proteins encoded by participating genes. Proteins encoded by *APEX1* and *RPAP1* genes were not in direct cross talk but were mediated by a third protein, cyclin O protein (CCNO). Similarly, protein-protein interactions between proteins encoded by *MLH1* and *MDM2* genes were predicted to be mediated by cyclin G1 protein (CCNG1). It was noteworthy that CCNG1 was acting as a central molecule interacting with proteins encoded by the four genes involved in our two-way SNP-SNP interactions and the mediating *CCNO* gene. Further, protein-protein interactions facilitated by CCNG1 and GATA zinc finger domain containing 2A (GATAD2A) proteins were also predicted to mediate interactions among proteins encoded by *MDM2*, *MLH1, MBD2* and *BRCA2* genes. We were limited in our ability to draw any finer conclusions since the number of genes considered for the study does not represent a comprehensive view of all DNA repair/metabolism genes on the human genome. To-date, the total number of human DNA repair genes annotated is around 130 [44]. The summarized work here merely provides a previously unexplored rationale and may generate hypothesis to test under various experimental designs, both for genetic and biological relevance beyond the provided statistical paradigm. Since the GENEMANIA network analysis is based on experimentally determined functional relationships, it is reasonable to speculate that both the two-way and multi-way SNP-SNP interactions and the known biological relationships among the proteins encoded by corresponding genes suggest possible cross talk and convergence of DNA repair, modification and metabolism pathways contributing to breast cancer etiology; this is consistent with the polygenic nature of complex diseases. The effect sizes from the SNP-SNP interactions were consistent with the predicted polygenic models (small but finite effect sizes from diverse gene/loci) and findings from GWASs to-date (ORs<1.5). Since a majority of breast cancer risk is explained by the intersection of life style factors with genetic predisposition, future studies may benefit by considering these additional risk factors to comprehensively account for the breast cancer risk in populations. However, caution should be exercised while interpreting the results from our interaction analyses until independent replication by other research groups could as well demonstrate the validity of statistical approaches to this emerging discipline of epistasis as a model to explain the additional missing heritable components of genetic risk.

In summary, we demonstrated both two-way and multi-way SNP-SNP interactions contributing to breast cancer risk, among candidate SNPs related to DNA repair, modification and metabolism pathway genes. The interactions were not previously reported and were mostly among the SNPs with weak but reproducible single-locus effects. Our results suggest SNP-SNP interactions among SNPs with weak but reproducible single-locus effects in a typical multi-stage GWAS or candidate-gene studies may identify cross talk among members of multiple cancer-related pathways, and help account for the heritability for complex diseases.

## Supporting Information

Table S1 **Associations of the six putative breast cancer susceptibility loci in stage 3 and in combined stages.**
(XLSX)

Table S2 **Eleven candidate DNA repair SNPs and their associations with breast cancer susceptibility in 2,720 breast cancer cases and 4,505 apparently healthy controls.**
(XLSX)

Text S1 **Methodology and pertinent discussion for single-locus association analyses of the 17 SNPs considered in the current study.**
(DOCX)
