# Supplementary material for: Assessing SNP-SNP Interactions among DNA Repair, Modification and Metabolism Related Pathway Genes in Breast Cancer Susceptibility
Source: PLoS One. 2013 Jun 3;8(6):e64896. doi: 10.1371/journal.pone.0064896 (PMC3670937; doi:10.1371/journal.pone.0064896)
Supplement: Text S1 — Methodology and pertinent discussion for single-locus association analyses of the 17 SNPs considered in the current study. (DOCX) [file pone.0064896.s003.docx]

**Supplementary Text 1: Methodology and pertinent discussion for single-locus association analyses of the 17 SNPs considered in the current study**

We initially investigated the single-locus effects of the 17 SNPs considered for potential epistatic effects. Both allelic and genotypic single-locus effects of SNPs for breast cancer were determined. Associations of SNPs with breast cancer susceptibility were evaluated with correlation/trend tests with one degree of freedom (d.f.). The magnitude of allelic and genotypic effects of the six putative breast cancer susceptibility SNPs (our previous work) [26] were estimated using unconditional logistic regression and reported as odds ratios (ORs) and corresponding 95% confidence intervals (CIs). Cases and controls from all three independent stages were pooled together and combined analysis was conducted. BMI was included as covariate in the logistic regression models: we, therefore, report BMI-adjusted ORs, 95% CI and *P* values of the six susceptibility SNPs and the additional 11 DNA repair SNPs.

Corrections for multiple comparisons were performed by conventional *P*=0.05/number of single-locus tests. Correlation/trend tests were performed using SNP and Variation Suite v7.6.11 (Golden Helix, Inc., Bozeman, MT, [www.goldenhelix.com](http://www.goldenhelix.com)) [45]. The observed and adjusted allelic and genotypic ORs and 95% CI and adjusted *P* values were estimated using logistic models available in PLINK [37]. R.2.15.1 was used for general statistical analyses.

The results from the single-locus tests and combined analyses across three stages of the study are briefly summarized - In stage 3, associations of the six SNPs (from our previous work) [26] showed consistency in terms of the magnitude and direction of associations in both allelic and genotypic tests but did not show statistical significance at *P*<0.05 (**Table S1**). However, in combined analysis, all SNPs demonstrated weak single-locus effects for breast cancer at *P*<0.05, and were independent of BMI. The three *MBD2* SNPs showed more statistically significant associations than the rest and were significant even after correction for multiple comparisons (combined unadjusted *P*<2.7 x 10^-3^ and adjusted *P*<1.6 x 10^-2^) (**Table S1**). Four DNA repair SNPs (*MLH1*-rs1799977, *MDM2*-rs769412, *BRCA2*-rs1799943 and *XRCC1*-rs25487) showed significant associations with breast cancer susceptibility at *P*<0.05 (**Table S2**). Of these, *MLH1*-rs1799977 and *MDM2*-rs769412 conferred reduced risk of breast cancer with allelic and genotypic ORs ranged from 0.76 to 0.94 while *BRCA2*-rs1799943 and *XRCC1*-rs25487 indicated risk-elevating effects with allelic and genotypic ORs ranged from 1.07 to 1.31. These weak single-locus effects were independent of BMI. *BRCA2*-rs1799943 showed a more statistically significant association and was significant even after correction for multiple comparisons, with per-allele ORs and 95% CI= 1.15 [1.06-1.25], OR_heterozygote_ and 95% CI= 1.15 [1.03-1.29], OR_homozygote_ and 95% CI= 1.31 [1.07-1.61] and *P*=9.3 x 10^-4^, adjusted for BMI.
